# Clinical indications associated with new opioid use for pain management in the United Kingdom: using national primary care data

**DOI:** 10.1097/j.pain.0000000000003402

**Published:** 2024-10-24

**Authors:** Carlos Raul Ramirez Medina, Max Lyon, Elinor Davies, David McCarthy, Vanessa Reid, Ashwin Khanna, Meghna Jani

**Affiliations:** a Centre for Epidemiology Versus Arthritis, Centre for Musculoskeletal Research, The University of Manchester, Manchester, United Kingdom; b The University of Manchester Medical School, Manchester, United Kingdom; c Manchester Royal Infirmary, Manchester University Foundation Trust, Manchester, United Kingdom; d Manchester and Salford Pain Centre, Salford Royal Hospital, Northern Care Alliance, Salford, United Kingdom; e NIHR Manchester Biomedical Research Centre, Manchester University NHS Foundation Trust, Manchester Academic Health Science Centre, Manchester, United Kingdom; f Department of Rheumatology, Salford Royal Hospital, Northern Care Alliance, Salford, United Kingdom

**Keywords:** Opioid, Indications, Electronic health records, Primary care, Musculoskeletal, Pain

## Abstract

Prescription opioids for noncancer pain in the United Kingdom have increased over the past 2 decades, alongside associated harms. Policies addressing opioid prescribing must be tailored to individual patient needs with specific disease systems. The aim of this study was to evaluate clinical conditions associated with new opioid initiation in noncancer pain using nationally representative UK data. Primary care electronic health records from January 1, 2006, to September 31, 2021, were used from the Clinical Research Practice Datalink to identify incident opioid prescriptions. Patient histories were reviewed using code lists for opioid-related conditions with a 5-year look-back for chronic conditions and a 1-year look-back for surgical indications before opioid initiation. In total, 3,030,077 new opioid use episodes in 2,027,402 patients were identified, with 61% being women, 77% aged 45 years and older, and 48% from the highest deprivation quintile. Ten systems associated with opioid initiation were identified, which were not mutually exclusive, as patients could have opioids prescribed for multiple indications. The most common were musculoskeletal (80.8%), respiratory (57.6%), infections (30.4%), trauma/injury (20.4%), neurology (19.9%), and postsurgical indications (5.5%). Osteoarthritis (60.7%) and low back pain (41.0%) were the most frequent musculoskeletal conditions. Orthopedic surgeries accounted for 41.2% of all postsurgical indications. This is the first study in the United Kingdom evaluating large-scale national data to assess indications associated with opioid initiation. Nearly 3 quarters of new opioid prescriptions for noncancer pain were in patients with musculoskeletal conditions, often for conditions with limited evidence for opioid efficacy. These findings could inform targeted interventions and future policies to support nonpharmacological interventions in the most common conditions where opioid harms outweigh benefits.

## Abstract 2

Supplemental Digital Content is Available in the Text.

First, UK study on national data evaluates opioid initiation, finding over 80% of new prescriptions linked to musculoskeletal conditions.

## 1. Introduction

The opioid crisis in North America,^24^ recognised as a public health emergency, has drawn attention to similar concerns in many Western countries.^1,24^ In the United Kingdom, opioid prescriptions have more than doubled over the past 2 decades,^3,13^ in parallel with a 48.9% increase in opioid-related hospitalisations from 2008 to 2018.^8^ In 2021, opioids were involved in nearly half of all UK drug poisoning deaths (45.7%, 2219 fatalities).^5^ High levels of opioid prescribing have also raised concerns among policy makers and regulators including national reviews by Public Health England^40^ and the Medicines and Healthcare Regulatory Agency.^21^

Opioids have long been prescribed to relieve acute pain. However, for managing chronic pain, the National Institute for Health and Care Excellence,^27^ along with the Royal College of Anaesthetists,^6^ advise against the initiation of opioids. They advocate for alternatives such as nonpharmacological interventions and nonopioid drugs.^27^ Despite growing awareness among healthcare professionals and the general public about the risks associated with their use, opioids are still being prescribed for chronic noncancer pain due to a diverse set of challenges: limited therapeutic options,^39^ biased beliefs about efficacy,^19^ and the preferences of both patients and physicians.^34^ Previous research from North America suggests that opioids remain a common prescription for several acute and chronic pain conditions, some of which may be more benign, including back pain, surgical recovery, dental,^32^ and gynaecological pain.^30^ The initiation and prescribing characteristics of opioids vary depending on the clinical indication.^36^ Targeted speciality-specific interventions and tailored opioid prescribing strategies require a clear understanding of how different clinical indications influence the initiation of opioid use. However, much of the existing research has been based on small samples^18,26,30,35,36^ and in populations with very different cultural and prescribing contexts and motivations than the United Kingdom and Europe. While there is increasing awareness of individual and prescribing factors associated with long-term opioid use in noncancer pain, the diverse reasons for prescribing opioids have not yet been assessed.^13^

The aim of this study was to evaluate the apparent clinical indications associated with new opioid initiation in noncancer pain using nationally representative UK primary care data. Importantly, the objective was not to assess the appropriateness of opioid prescriptions but to increase awareness of the possible reasons for opioid initiation, including both chronic and acute pain conditions.

## 2. Methods

### 2.1. Study design and data source

We conducted a retrospective observational study from the 1st of January 2006 to the 31st of September 2021 using the Clinical Practice Research Datalink (CPRD) Aurum, a database of anonymised UK primary care electronic health records. General practice is the first line of contact for patients presenting with medical needs in the United Kingdom and are responsible to community-based care and referral to secondary care. CPRD is a widely used resource^33^ and one of the largest research databases of longitudinal primary care records in the world and contains information from >14 million registered patients, including prescriptions and clinical data such diagnoses.^12^ CPRD provides pseudonymised patient level data from a network of general practices across the United Kingdom. Approximately 16% of general practices (1345 of 8178)^2^ in the United Kingdom contribute to CPRD Aurum through an opt-in system that has been operational for over 30 years. Over 1000 research studies examining a wide variety of health outcomes have been published in peer-reviewed journals using data from CPRD.^12^ The database has undergone several validation assessments to evaluate correctness and completeness, finding coded diagnoses generally reliable.^31^ In addition, CPRD Aurum data were linked to English Index of Multiple Deprivation (IMD) 2019 and to the Hospital Episode Statistics database to retrieve ethnicity information.

### 2.2. Study population

This study involved adult individuals who were aged 18 years and older, with newly prescribed opioids, and without a history of cancer. Patients were considered new opioid users if they had a 2-year opioid-free interval before a new incident prescription. Exclusion criteria included individuals with any cancer history identified through mapped Read codes within the 5 years preceding the index date and those below the age of 18 years at the index date. Only practices with “up to standard” follow-up were included in this study to select research-quality patients and periods of quality in data recording.^12^

**Figure 1.**
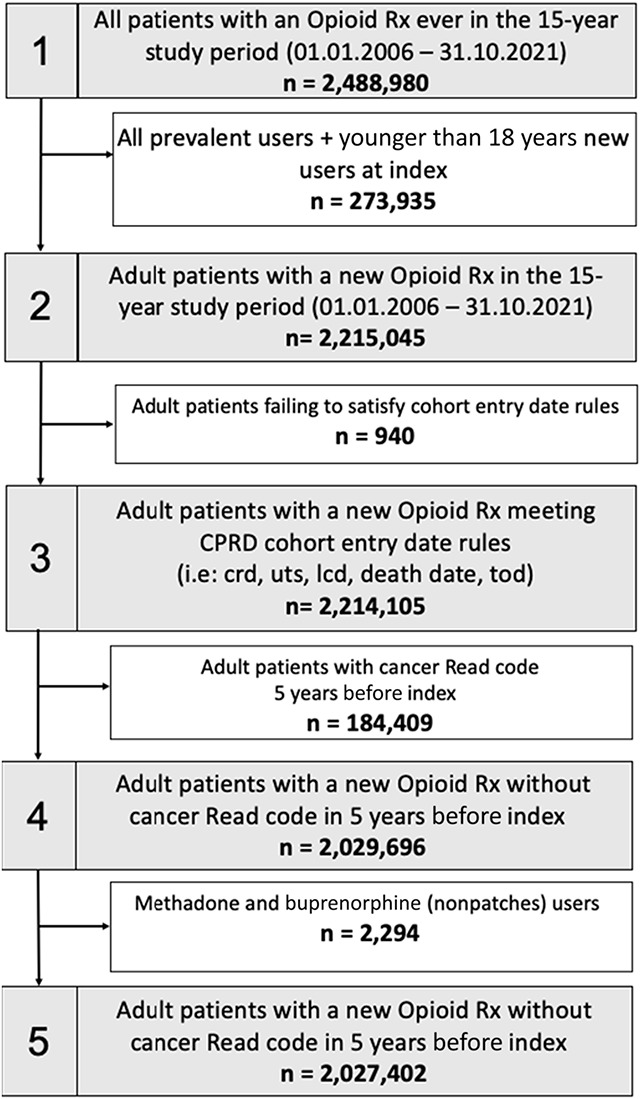
Study cohort derivation diagram. Throughout the 15-year study window, 3,030,077 new opioid episodes were identified for 2,027,402 unique patients. Patients were considered new opioid users if they had a 2-year opioid-free interval before a new incident prescription. crd, date of the patient's current period of registration with the practice began; lcd, date of the last collection of data for the practice; hes, hospital episode statistics; tod; date of the patient transferred out of the practice; uts, up to standard/date at which the practice data were deemed to be of research quality.

To identify opioid prescriptions, we used a curated comprehensive code list covering all prescription opioids, including various formulations and routes of administration. In our study, users prescribed formulations of buprenorphine other than transdermal patchs (commonly prescribed for pain) were excluded due to their primary use in opioid use disorder treatment rather than pain management. In addition, individuals prescribed methadone at the index date were excluded as it is predominantly used for treating opioid addiction in the United Kingdom (Fig. 1).

### 2.3. Identification of clinical indications

A comprehensive list of 53 clinical indications for opioid use was compiled in consultation with UK-based clinicians with expertise in pain management general practice, specialist pharmacy, general medicine, and rheumatology (M.J., M.L., A.K., V.R., D.M.) and following evaluation of the existing scientific literature. Indications were categorised into 10 distinct medical systems (ie, Rheumatic and Musculoskeletal Diseases, Respiratory, Infections, Trauma/Injury, Neurology, Major Surgery, Gynaecological reasons, Gastrointestinal, Dental, and Haematological). The methodology used for generating the code lists needed for this study is based on previous studies^11^ and summarised in (Supplementary Figure 1, http://links.lww.com/PAIN/C144).

Clinical observations in CPRD Aurum are recorded using a mixture of Read-2, SNOMED, and local Egton Medical Information Systems codes. We created specific medical code lists for each clinical indication, which were then used to identify records of these indications in a cohort of new opioid users without cancer. For surgical indications, we reviewed records from 1 year before the index date, given that procedures are short-term and would be coded as such during the time of the event. For chronic conditions, we extended this look-back period to 5 years before the index date as such conditions would not be expected to be coded repeatedly on a yearly basis once diagnosed. As sensitivity analyses, clinical diagnoses registered in the patient file on the day of new opioid prescription and using a 1-year look-back period for chronic conditions were examined.

### 2.4. Prescription and patient characteristics

Patient demographic characteristics were evaluated including age, sex, and the IMD stratified by each system. We analysed prescription details at initiation for all patients, including the estimated daily dose dispensed in morphine milligram equivalents (MMEs) per day. Prescription data were prepared using a Drug Preparation Algorithm from the “drugprepr” R package,^15^ which our team developed for transparent data preparation. The MME/day was defined as the daily dose for each prescription multiplied by the equivalent analgesic ratio as specified by the US Centers for Disease Control and Prevention. Conversion guidelines followed the US Centers for Disease Control and Prevention standards.^4^ Implausible dose values and missing values were handled transparently by the Drug preparation algorithm.^15^ Assumptions are detailed in (Supplementary Methods, http://links.lww.com/PAIN/C144).

Descriptive statistics were used to evaluate the indications, demographics, and prescribing characteristics for each system. As may be the case in clinical practice, systems were not mutually exclusive as patients could have an opioid prescribed for multiple indications, therefore were able to overlap. We reported the presence of any missing patient data as observed. Proportions of each medical system were reported in relation to the total of 10 distinct medical systems. Visualisation for the quantitative analysis of sets and their intersections were prepared using a range of visualisation techniques in Python, Microsoft Power BI, and R. Data were cleaned and processed using Stata v13.1 (StataCorp LLC, College Station, TX).

The study was approved by the CPRD's Independent Scientific Advisory Committee (approval numbers: 23_002658 and 20_000143). This study is reported as per the Strengthening the Reporting of Observational Studies in Epidemiology guideline (S1 Strengthening the Reporting of Observational Studies in Epidemiology Checklist).

## 3. Results

Throughout the 15-year study window, a total of 3,030,077 new opioid episodes were identified in CPRD Aurum for 2,027,402 unique patients (Fig. 1).

**Figure 2.**
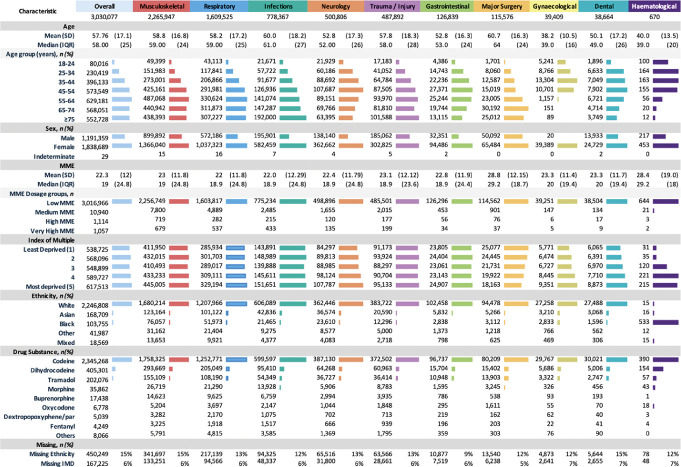
Patient characteristics of opioid-naïve use by clinical indication system. Proportions are presented as percentage of nonmissing data. Some patients had missing data for certain variables, which is reported at the end of the table. Other drug substances include: diamorphine, meptazinol, pethidine, tapentadol, dipipanone, alfentanil, hydromorphone, pentazocine, papaveretum, and dextromoramide tartrate. Opioid Dosage: Low MME defined as <50 MME/day. Medium MME (50-119 MME/day), High MME (120-199 MME/day), Very High MME (≥200 MME/day). Full Table with percentages can be found in the Supplementary Table 1 (http://links.lww.com/PAIN/C144). IQR, interquartile range; MME, morphine-milligram equivalent.

The mean age of the patients in our study cohort was 57.7 years (SD: 17). Most new opioid users were older than 55 years (57.8%), with the largest proportion within the 55 to 64 years age band (20.8%), while the least represented age group was 18 to 24 years (2.6%). Female patients constituted 61% of the cohort. Of the 5 IMD quintiles, 43% of patients were in the most deprived quintiles (21% quintile 4, 22% quintile 5). In this study, 87% of patients were White or Caucasian ethnicity, with smaller subpopulations of other ethnicities (Fig. 2).

A total of 53 clinical indications within 10 systems were identified. The most common systems among patients were musculoskeletal (80.8%), respiratory (57.6%), infections (30.4%), trauma/injury (20.4%), neurology (19.8%), postsurgical indications (5.5%), and gastrointestinal (5.2%; Table 1).

**Table 1:** Clinical indications of opioids for new opioid patients by systems. Counts by unique patients in the 15-year study.

System, n (%)		Total unique patients	2,027,402	100%
		Indications	n	(%)
Musculoskeletal	1,639,142 (80.8%)	Osteoarthritis	1,231,490	60.74%
		Low back pain	831,636	41.02%
		MSK (noninflammatory) bursitis, rotator cuff, tendonitis	292,620	14.43%
		Osteoporosis	96,857	4.78%
		Gout	87,421	4.31%
		Rheumatoid arthritis	49,959	2.46%
		Fibromyalgia	48,748	2.41%
		Psoriatic arthritis	11,287	0.56%
		Systemic lupus erythematosus	6761	0.33%
		Ankylosing spondylitis	6686	0.33%
		Myositis	1945	0.10%
Respiratory	1,168,589 (57.6%)	Respiratory infections	920,511	45.40%
		Cough	702,733	34.66%
		Respiratory (noninfective)	98,901	4.88%
		Pulmonary fibrosis	5962	0.29%
Infections	616,452 (30.4%)	Infections (most commonly UTI/cellulitis/otitis media)	615,211	30.34%
		HIV	1754	0.09%
Trauma/injury	413,005 (20.4%)	Trauma (including sprains, sprains, and dislocations)	228,298	11.26%
		Fractures	220,296	10.87%
Neurology	401,834 (19.8%)	Headaches (including migraine)	366,193	18.06%
		Neuropathic pain	33,813	1.67%
		Demyelinating conditions (including GBS, multiple sclerosis)	15,422	0.76%
		Somatoform	181	<0.01%
Major surgery	111,269 (5.5%)	Total knee replacement	30,930	1.52%
		Total hip replacement	17,313	0.85%
		Hernia repair	12,230	0.60%
		Hysterectomy	10,919	0.54%
		Caesarean section	9380	0.46%
		Cholecystectomy	7955	0.39%
		CABG	5783	0.29%
		Vasectomy	4033	0.20%
		Valve replacement	3570	0.18%
		Appendectomy	3520	0.17%
		Prostatectomy	2661	0.13%
		Laparoscopy	2152	0.11%
		Limb amputations	1466	0.07%
		Rotator cuff surgeries	1304	0.06%
		Carotid endarterectomy	666	0.03%
		Aortic aneurysm repair	653	0.03%
		Resection of bladder tumour	472	0.02%
		Mastectomy	310	0.02%
		Lobectomy	244	0.01%
		Resection of prostate (including TURP)	206	0.01%
		Lumpectomy	179	<0.01%
		Breast reconstruction	125	<0.01%
		Aortic root replacement	92	<0.01%
		Pneumonectomy	12	<0.01%
Gastrointestinal	104,951 (5.2%)	IBS	87,980	4.34%
		IBD	16,979	0.84%
		Chronic pancreatitis	1414	0.07%
Dental	34,744 (1.7%)	Dental pain	34,744	1.71%
Gynaecological reasons	33,927 (1.7%)	Including: (1) dysmenorrhoea/Endometriosis, (2) ovarian cysts, fibroids/leiomyoma (3) PID, cervicitis (4) adhesions and hydrosalpinx	33,927	1.67%
Haematological	519 (0.0%)	Sickle cell	519	0.03%

CABG, Coronary artery bypass grafting; HIV, human immunodeficiency virus; IBD, inflammatory bowel disease; IBS, irritable bowel syndrome; GBS, Guillain-Barré syndrome; MSK, musculoskeletal; PID, pelvic inflammatory disease; TURP, transurethral resection of the prostate; UTI, urinary tract infection.

The most common musculoskeletal indications among unique patients who were new opioid users were osteoarthritis (60.7%), low back pain (41.0%), musculoskeletal noninflammatory conditions (14.4%), and osteoporosis (4.8%). Musculoskeletal noninflammatory conditions included rotator cuff syndrome, bursitis, epicondylitis, tendonitis, strain injury, and impingement syndrome. Inflammatory arthritis, including rheumatoid arthritis, systemic lupus erythematosus, psoriatic arthritis, and ankylosing spondylitis, accounted for 3.6% of our cohort (Table 1).

The respiratory system included patients with indications for respiratory infections (45.4%), cough (34.7%), and noninfective respiratory issues (4.9%), such as chronic obstructive pulmonary disease, chronic bronchitis, bullous emphysema, and pulmonary fibrosis. Trauma/injury accounted for 20.4% (413,005 patients) of the cohort, with specific indications such as sprains, strains, dislocations (11.3% of total cohort), and fractures (10.9% of total cohort). Neurological indications were observed in 19.8% (401,834 unique patients), primarily comprising headaches (18.0% of total cohort) and neuropathic pain (1.7% of total cohort).

**Figure 3.**
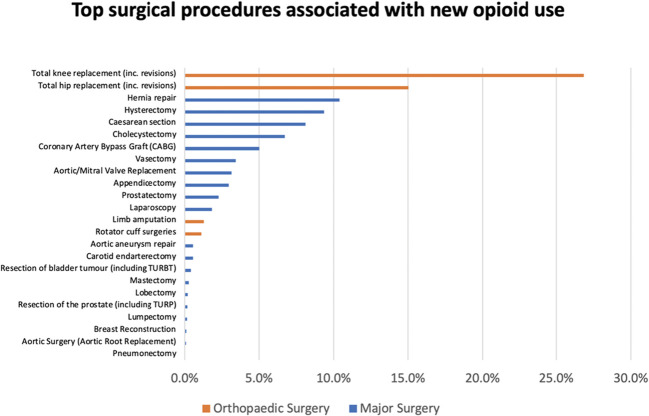
The most common surgical procedures associated with new opioid use by groups (orthopedic surgery and major surgery).

Individuals who underwent major surgery within 1 year before the opioid prescription represented 5.5% (111,269 unique patients) of the cohort, with orthopedic surgeries such as total knee replacement (26.9% of all surgical procedures) and total hip replacements (15.0% of all surgical procedures) being the most common (Fig. 3).

Other major surgeries spanned various specialties including general surgery, and obstetrics and gynaecology. Notable among these were hernia repairs (10.4% of surgical procedures), hysterectomies (9.4% of surgical procedures), and caesarean sections (8.1% of surgical procedures). On the lower end, surgeries such as pneumonectomy and aortic root replacement constituted less than 0.1% of cases of surgical procedures.

Gastrointestinal conditions, such as irritable bowel syndrome, inflammatory bowel disease, and chronic pancreatitis, were found for 5.2% of our cohort (n = 104,951 patients). Gynaecological issues represented 1.7% of the study cohort (n = 33,927 patients), including dysmenorrhea, endometriosis, ovarian cysts, fibroids, cervicitis, adhesions, and hydrosalpinx.

A minority of new opioid users were reported to have dental problems (1.7%) including caries, bridge work, infections, abscesses, fractures, and extractions or haematological conditions (0.3%), notably sickle cell disease. In 12% of new opioid users (239,495 individuals), a specific clinical indication was not discovered using the 5-year period for chronic conditions and 1-year period for surgical procedures.

The most common IMD quintile was the most deprived group accounting for 22% of our cohort. This trend was consistent across all clinical systems with the exception of major surgery, where the less deprived quintile was more common, at 23% (Fig. 2). Ethnicity demographics showed the White Caucasian as the predominant group in most clinical systems, except for the haematological system, where the Black Ethnicity was more common (90%). Overall, women constituted most patients (61%) across all medical systems, except in major surgery, where the distribution was more balanced between male patients and female patients (43% male vs 57% female). In the gynaecological clinical system, the predominant age groups were 35 to 44 and 45 to 54 years, while for sickle cell haematological cases, younger patients (<35 years old) were more common.

In terms of daily MME at initiation, a predominance of low-dose opioid prescriptions (defined as <50 MME/day) was found across the cohort (n = 3,016,966 new opioid episodes, 99%). Patients who underwent major surgery or experienced trauma/injuries, as well as those requiring dental interventions and haematological patients, exhibited higher mean and median MME/day doses in comparison with other clinical categories (Table 1). Codeine was found to be the most common prescribed opioid (77.4% of all new prescriptions), followed by dihydrocodeine (13.4%), tramadol (6.7%), and morphine (1.2%). These patterns were consistent across all clinical systems examined. In major surgery, prescription rates of tramadol, morphine, and oxycodone were slightly higher in comparison with their usage in other clinical settings.

**Figure 4.**
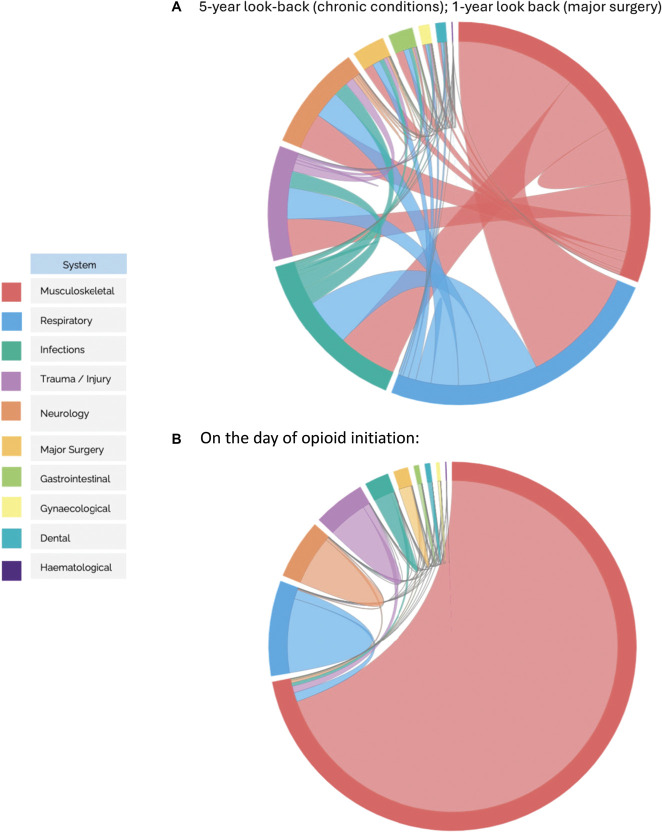
Overlaps between Clinical Indication Systems. These chord diagrams visualise the intersections between the top 10 clinical systems observed among new opioid users with (A) a 5-year look-back period for chronic conditions and 1-year look-back period for major surgery and (B) the same day of the initial prescription, with emphasis on the 2-way overlaps between medical systems. The diagram highlights the relationships and overlaps between these clinical systems through chords connecting each pair of entities. The segments within the outer circle (also called arcs) are proportional to the percentage of opioid use for each system. Chords represent the overlaps between the systems. The width of the chords represents how large the overlap between each system is. Musculoskeletal conditions were the most common clinical indication system among new opioid users.

Patients may receive opioid prescriptions for multiple reasons, allowing for overlaps between different clinical systems. The largest overlap was observed between the musculoskeletal and respiratory systems, involving 963,416 unique patients (31.6%) as shown in (Fig. 4), followed by an overlap between musculoskeletal and infections systems (n = 505,411 patients).

**Figure 5.**
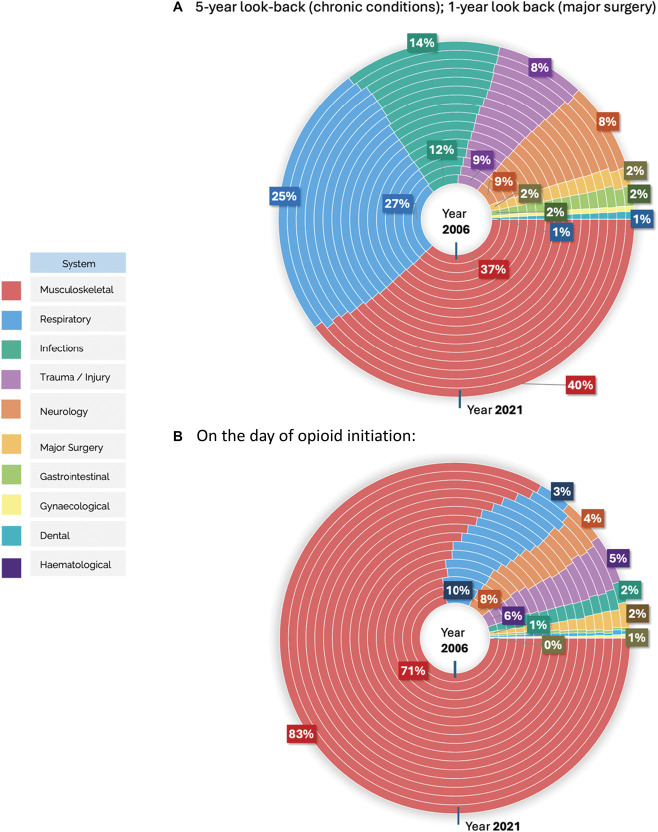
Changes in proportions of clinical indications systems among new opioid users from 2006-2021, relative to the total clinical indication systems identified each year. These nested pie charts show how much each clinical system contributed to the overall pool of clinical systems for that year, based on (A) a 5-year look-back period for chronic conditions and a 1-year look-back period for major surgeries, and (B) the day of the initial opioid prescription. Each concentric ring corresponds to a specific year, with the innermost ring representing 2006 and the outermost, 2021. For more details on how these percentages were calculated and for the percentages representing the proportion of each clinical system relative to the total number of opioid users each year, see Supplementary Tables 4 and 5 (http://links.lww.com/PAIN/C144).

In addition, we conducted a sensitivity analysis focussing on conditions recorded on the same day as the initial opioid prescription, providing a more immediate and relevant snapshot of clinical indications. This analysis revealed that musculoskeletal conditions registered on the day of new opioid initiation were overwhelmingly the most common compared with the other 9 medical systems (Fig. 5), with low back pain being the most frequently recorded condition within this category. In our primary analysis, which included a 5-year lookback period, clinical systems such as respiratory, neurology, and trauma had a higher proportion of cases, balancing out with the musculoskeletal conditions. The on-day analysis clearly shows that musculoskeletal conditions were the predominant group, as did the one-year sensitivity analysis look back period (Supplementary Tables 2 and 3, http://links.lww.com/PAIN/C144).

Musculoskeletal conditions showed an increase in proportions relative to the other 9 clinical systems during the 15-year study, while respiratory conditions demonstrated a decline. Neurological conditions emerged as the third most frequent category, followed by trauma injuries and infections, which contrasts with the earlier findings from the 5-year look-back period.

## 4. Discussion

In this comprehensive nationwide study, which included 2,027,402 patients (3,030,077 new opioid episodes), we observed a wide range of likely indications for new opioid initiation. Musculoskeletal clinical indications were found to be the most common system among new opioid users in primary care, followed by respiratory conditions and infections. The most common surgical indications were orthopaedic surgeries such as total knee and hip replacements, followed by hernia repair.

Our study indicates that opioids are commonly prescribed in patients with musculoskeletal conditions, particularly osteoarthritis and low back pain, despite limited scientific evidence supporting their effectiveness. For chronic low back pain and osteoarthritis, opioids offer no advantage over nonopioid medications and could potentially lead to more harms.^16,17^ Recent studies have shown that opioids are no more effective than nonopioid treatments and carry higher risks of adverse effects. For these conditions, opioids are less effective than nonsteroidal antiinflammatory drugs, with benefits diminishing over time, making their regular use for managing pain inadvisable.^16,17^

The high proportion of new opioid users in UK primary care with musculoskeletal clinical indications (eg, chronic pain, osteoarthritis) is relevant, given the ageing population of United Kingdom.^7^ Similar findings were observed in Spain, where musculoskeletal pain was the most common cause of opioid initiation, constituting 65.1% of such cases.^9^ Interestingly, this study also found a pattern of opioid use for mild respiratory conditions such as cough, which could also explain the association of new opioid prescriptions with respiratory conditions in our cohort. This raises concerns about potential inappropriate opioid prescribing practices in the United Kingdom that merit further investigation.

A Finnish study reported musculoskeletal disorders as the main reason for long-term opioid use in care home residents, with neuropathic pain also common.^25^ Unlike our findings, buprenorphine was the most prescribed opioid in their study, likely due to and varied preferences for initial opioids across different countries and jurisdictions.^14^

A Canadian study found acute pain, including dental, postsurgical, and trauma-related pain, to be the most common indication for new opioid prescriptions.^10^ These results differ from our study, most likely because acute care hospital prescriptions and out-of-hours urgent prescribing are not routinely recorded in the primary care prescribing data in the United Kingdom.^29^ In addition, the Canadian study used a hierarchical approach to identify clinical indications, which did not account for overlaps or simultaneous presence of multiple clinical indications. In our study, most dental-related data were not available as CPRD is a primary care database, which does not collect data from dental practices that are a combination of private and public funded in the United Kingdom. However, the proportion of prescriptions prescribed by physicians in the Canadian study is similar to that reported in this article. Previous investigation into dentist opioid prescription in the United Kingdom in 2016 demonstrated relatively reserved prescribing of these medications, particularly in comparison with their US counterparts, with only 28,082 dental-related opioid prescriptions being issued in 2016.^38^ Therefore, it is probable that opioid prescribing for dental indications differs between the United Kingdom and North American countries. However, the most common nonsurgical indications associated with longer duration of opioid prescription were reported as back pain joint and muscle pain, which further emphasises the prolonged opioid burden in these chronic conditions.

Research in the United States revealed that diagnoses such as osteoarthritis, joint, and back disorders were found to be the most important drivers of opioid prescriptions among patients with obesity.^37^ These findings mirror the results of our study, further underscoring the significant burden of musculoskeletal conditions. Obesity is a condition strongly associated with osteoarthritis, causing joint damage through biomechanical stress and inflammation.^41^ This is likely to become a growing problem in the United Kingdom, with 35% of adults projected to be obese by 2030.^28^ While in this study, we did not include an analysis of obesity among patients, it would be beneficial to consider additional measures to reduce the health burden of obesity within the United Kingdom, alongside efforts aimed at reducing opioid prescribing.

The substantial overlap between clinical systems highlights a complex clinical landscape, suggesting that patients with comorbidities or multifaceted pain conditions may receive opioid prescriptions to manage their symptoms. This emphasizes the importance of adopting a holistic approach to pain management, as well as a careful evaluation of the risks and benefits of opioid therapy in patients with multiple clinical indications.

### 4.1. Strengths of the study

To the best of our knowledge, this study represents the first comprehensive, nationwide research into the clinical reasons behind the initiation of opioid prescriptions in primary care settings within the United Kingdom. With a look-back period of 16 years, we were able to analyse one of the largest data sets to date and better understand the nature of opioid prescribing over a considerable time period. Comprehensive code lists for identification of clinical systems were developed in consultation with UK-based clinicians with expertise in pain management, specialist pharmacy input, general medicine, and rheumatology. Such collaboration ensures that our analysis is grounded in an understanding of pain-related conditions in the United Kingdom. Furthermore, the majority of opioid prescribing occurs in primary care, and, by using these data, we have been able to further understand the patterns of prescribing within this healthcare sector with a representative sample.

### 4.2. Limitations of the study

Our study exhibits several limitations that warrant consideration. In the United Kingdom, general practitioners in primary care are not required to record a formal indication for every drug prescription. This is reflected in the data set we used, CPRD Aurum, which relies on routinely collected primary care data. CPRD Aurum does not capture specific justifications for prescribing drugs. The closest alternatives we have are clinical codes entered by healthcare professionals into the patient record. However, these codes are not always documented at the time of each new prescription, particularly for chronic conditions. This is not a limitation of the CPRD data set itself but rather reflects how information is routinely recorded in UK clinical practice. Within the constraints of CPRD, this approach offers the most accurate estimation possible and aligns with methods used in other studies.^9,30^ Our sensitivity analysis focussing on conditions documented on the same day as the initial opioid prescription, aimed to provide additional information on the clinical reasons behind opioid prescribing.

In the main analysis, for 12% of users within our cohort, we were unable to identify a clinical indication from our code lists within our defined time frames. We observed instances where individuals exhibited symptoms rather than formal diagnoses, making it challenging to categorise them into specific indication groups. This could be explained by the approach used, which set look-back period of 5/1 years and, therefore, potentially not capturing the initial diagnosis. However, a longer look-back period would have further weakened the association between the opioid prescription and diagnosis.

It is important to note that our analysis focused solely on opioids prescribing within primary care settings. In the United Kingdom, opioids are controlled substances, and their sale is tightly regulated. We acknowledge that over-the-counter (OTC) pain-relief medications containing low dose codeine, or dihydrocodeine can still be purchased without a prescription in the United Kingdom. However, these OTC opioids are not a major contributor to overall opioid use and represent a minority of opioid consumption in comparison with what is prescribed.^20^ National UK efforts from the Medicines and Healthcare products Regulatory Agency have included reducing the pack size of what is available OTC and more recently making Codeine Linctus prescription only.^22^

In addition, our use of CPRD Aurum has inherent limitations, including potential missing data. Within Egton Medical Information Systems clinical systems, healthcare professionals have the flexibility record clinical observations using free text rather than structured coding. CPRD does not release free text due to information governance restrictions, to protect confidential patient information but could have provided more insight into why those without an associated Read Code were prescribed an opioid.

### 4.3. Broader implications and impact on future policy

This study provides further insight into opioid prescribing in the United Kingdom and helps to identify groups that are more likely to be prescribed this class of drug. Specifically, it identifies musculoskeletal conditions such as osteoarthritis and low back pain as the most common musculoskeletal conditions behind opioid initiation, despite very little scientific evidence supporting their effectiveness in chronic noncancer pain.^16,17^ Recent findings indicate that opioids may offer little advantage over nonopioid treatments for these conditions and could pose greater risks.

By focussing on opioid initiation, this study underscores the gap between current prescribing practices and evidence-based guidelines, prompting a re-evaluation of prescribing behaviours. These findings can guide efforts to deprescribe and encourage clinical vigilance, focussed particularly on these groups to reduce inappropriate opioid prescribing.

## 5. Conclusions

This is the first study in the United Kingdom evaluating large scale national data to assess indications associated with opioid initiation. Almost 3 quarters of the new prescriptions of opioids for noncancer pain were in people with a diagnosis of a musculoskeletal condition. Orthopedic surgeries contributed to a third of all postsurgical indications. Understanding the opioid utilization patterns for these conditions is crucial, given the potential risks associated with long-term opioid use, including dependency and adverse effects. These findings could help prioritise efforts for targeted interventions in opioid prescribing, clinical vigilance, and future policy to support nonpharmacological interventions.

## Conflict of interest statement

All authors report no conflicts of interest regarding this manuscript.

The data used for this paper are available through The CRPD (https://www.cprd.com/, contact for data queries: enquires@cprd.com) for researchers who meet criteria for access to confidential data. And additional materials are included in the supplementary section, http://links.lww.com/PAIN/C144.

## Appendix A. Supplemental digital content

Supplemental digital content associated with this article can be found online at http://links.lww.com/PAIN/C144.

## References

[1] Ayoo K Mikhaeil J Huang A Wąsowicz M. The opioid crisis in North America: facts and future lessons for Europe. Anaesthesiol Intensive Ther 2020;52:139–47. 32419434 10.5114/ait.2020.94756 PMC10176520

[2] Clinical Practice Research Datalink. CPRD Aurum May 2022 (Version 2022.05.001) [Data set]. Clinical Practice Research Datalink, 2022. 10.48329/t89s-kf12.

[3] Curtis HJ Croker R Walker AJ Richards GC Quinlan J Goldacre B. Opioid prescribing trends and geographical variation in England, 1998–2018: a retrospective database study. Lancet Psychiatry 2019;6:140–50. 30580987 10.1016/S2215-0366(18)30471-1

[4] Dowell D Haegerich TM Chou R. CDC guideline for prescribing opioids for chronic pain—United States, 2016. JAMA 2016;315:1624–45. 26977696 10.1001/jama.2016.1464 PMC6390846

[5] Duke K Gleeson H MacGregor S Thom B. The risk matrix: drug-related deaths in prisons in England and Wales, 2015–2020. J Community Psychol 2023. doi: 10.1002/jcop.22989. 36601729

[6] Faculty of Pain Medicine of the Royal College of Anaesthetists. Opioids Aware. Vol. 2024. Royal College of Anaesthetists: Royal College of Anaesthetists in Collaboration with Public Health England, 2024. Available at: https://www.fpm.ac.uk/opioids-aware. Accessed September 13, 2024

[7] Fayaz A Croft P Langford RM Donaldson LJ Jones GT. Prevalence of chronic pain in the UK: a systematic review and meta-analysis of population studies. BMJ Open 2016;6:e010364. 10.1136/bmjopen-2015-010364 PMC4932255 27324708

[8] Friebel R Maynou L. Trends and characteristics of hospitalisations from the harmful use of opioids in England between 2008 and 2018: population-based retrospective cohort study. J R Soc Med 2022;115:173–85. 35114090 10.1177/01410768221077360 PMC9066666

[9] García-Sempere A Hurtado I Robles C Llopis-Cardona F Sánchez-Saez F Rodriguez-Bernal C Peiró-Moreno S Sanfélix-Gimeno G. Initial opioid prescription characteristics and risk of opioid misuse, poisoning and dependence: retrospective cohort study. BMJ Qual Saf 2023;33:13–23. 10.1136/bmjqs-2022-015833 PMC10804034 37414557

[10] Gomes T Men S Campbell TJ Tadrous M Mamdani MM Paterson JM Juurlink DN. Changing patterns of opioid initiation for pain management in Ontario, Canada: a population-based cross-sectional study. PLoS One 2022;17:e0278508. 36480526 10.1371/journal.pone.0278508 PMC9731435

[11] Graul EL Stone PW Massen GM Hatam S Adamson A Denaxas S Peters NS Quint JK. Determining prescriptions in electronic health care (EHR) data: methods for development of standardised, reproducible drug codelists. JAMIA Open 2023;6:ooad078. doi: 10.1093/jamiaopen/ooad078. 37649988 PMC10463548

[12] Herrett E Gallagher AM Bhaskaran K Forbes H Mathur R van Staa T Smeeth L. Data resource profile: clinical practice research datalink (CPRD). Int J Epidemiol 2015;44:827–36. 26050254 10.1093/ije/dyv098 PMC4521131

[13] Jani M Birlie Yimer B Sheppard T Lunt M Dixon WG. Time trends and prescribing patterns of opioid drugs in UK primary care patients with non-cancer pain: a retrospective cohort study. PLoS Med 2020;17:e1003270. 33057368 10.1371/journal.pmed.1003270 PMC7561110

[14] Jani M Girard N Bates DW Buckeridge DL Sheppard T Li J Iqbal U Vik S Weaver C Seidel J Dixon WG Tamblyn R. Opioid prescribing among new users for non-cancer pain in the USA, Canada, UK, and Taiwan: a population-based cohort study. PLoS Med 2021;18:e1003829. 34723956 10.1371/journal.pmed.1003829 PMC8601614

[15] Jani M Yimer BB Selby D Lunt M Nenadic G Dixon WG. “Take up to eight tablets per day”: incorporating free-text medication instructions into a transparent and reproducible process for preparing drug exposure data for pharmacoepidemiology. Pharmacoepidemiol Drug Saf 2023;32:651–60. 36718594 10.1002/pds.5595 PMC10947089

[16] Jones CMP O Day R Koes BW Latimer J Maher CG MacLachlan A Billot L Shan S Chung-Wei C. Opioid analgesia for acute low back pain and neck pain (the OPAL trial): a randomised placebo-controlled trial. Lancet 2023;402:304–12. doi: 10.1016/S0140-6736(23)00404-X. 37392748

[17] Krebs E Gravely A Nugent S Jensen AC DeRonne B Goldsmith E Kroenke K Blair MJ Noorbaloochi S. Effect of Opioid vs Nonopioid Medications on Pain-Related Function in Patients With Chronic Back Pain or Hip or Knee Osteoarthritis Pain: The SPACE Randomized Clinical Trial. JAMA 2018;319:872–82. doi: 10.1001/jama.2018.0899. PMC5885909 29509867

[18] Liu Y Logan JE Paulozzi LJ Zhang K Jones CM. Potential misuse and inappropriate prescription practices involving opioid analgesics. Am J Manag Care 2013;19:648–58. 24304213

[19] Manchikanti L Kaye AM Knezevic NN McAnally H Slavin K Trescot AM Blank S Pampati V Abdi S Grider JS Kaye AD Manchikanti KN Cordner H Gharibo CG Harned ME Albers SL Atluri S Aydin SM Bakshi S Barkin RL Benyamin RM Boswell MV Buenaventura RM Calodney AK Cedeno DL Datta S Deer TR Fellows B Galan V Grami V Hansen H Helm Ii S Justiz R Koyyalagunta D Malla Y Navani A Nouri KH Pasupuleti R Sehgal N Silverman SM Simopoulos TT Singh V Solanki DR Staats PS Vallejo R Wargo BW Watanabe A Hirsch JA. Responsible, safe, and effective prescription of opioids for chronic non-cancer pain: American Society of Interventional Pain Physicians (ASIPP) guidelines. Pain Physician 2017;20:S3–92. 28226332

[20] Medicines and Healthcare products Regulatory Agency. MHRA public assessment report. Codeine and dihydrocodeine-containing medicines: minimising the risk of addiction. London: MHRA, 2009.

[21] Medicines and Healthcare products Regulatory Agency. Opioid Expert Working Group meets at MHRA, 2019. Available at: https://www.gov.uk/government/news/opioid-expert-working-group-meets-at-mhra. Accessed September 13, 2024.

[22] Medicines and Healthcare products Regulatory Agency 2024. Codeine linctus to be reclassified to a prescription-only medicine because of risk of abuse and addiction. Availalble at: https://www.gov.uk/government/news/codeine-linctus-to-be-reclassified-to-a-prescription-only-medicine-because-of-risk-of-abuse-and-addiction. Accessed Septemeber 13, 2024.

[23] Meldrum ML. The ongoing opioid prescription epidemic: historical context. Am J Public Health 2016;106:1365–66. 27400351 10.2105/AJPH.2016.303297 PMC4940677

[24] Mordecai L Reynolds C Donaldson LJ de C Williams AC. Patterns of regional variation of opioid prescribing in primary care in England: a retrospective observational study. Br J Gen Pract 2018;68:e225–33. 29440012 10.3399/bjgp18X695057 PMC5819988

[25] Mörttinen-Vallius H Hartikainen S Seinelä L Jämsen E. The prevalence of and exact indications for daily opioid use among aged home care clients with and without dementia. Aging Clin Exp Res 2021;33:1239–47. 32613548 10.1007/s40520-020-01627-8 PMC8081682

[26] Mundkur ML Rough K Huybrechts KF Levin R Gagne JJ Desai RJ Patorno E Choudhry NK Bateman BT. Patterns of opioid initiation at first visits for pain in United States primary care settings. Pharmacoepidemiol Drug Saf 2018;27:495–503. 28971545 10.1002/pds.4322 PMC5880749

[27] National Institute for Health and Care Excellence. Chronic pain in over 16s: assessment of all chronic pain and management of chronic primary pain. London: NICE, 2021. 33939353

[28] OECD Obesity Update 2017. The Organisation for Economic Co-operation and Development (OECD). Available at: http://oecd.org. Accessed September 13, 2024.

[29] Okoli GN Myles P Murray-Thomas T Shepherd H Wong ICK Edwards D. Use of primary care data in research and pharmacovigilance: eight scenarios where prescription data are absent. Drug Saf 2021;44:1033–40. 34296384 10.1007/s40264-021-01093-9 PMC8297607

[30] Pasricha SV Tadrous M Khuu W Juurlink DN Mamdani MM Paterson JM Gomes T. Clinical indications associated with opioid initiation for pain management in Ontario, Canada: a population-based cohort study. PAIN 2018;159:1562–8. 29762260 10.1097/j.pain.0000000000001242 PMC6085129

[31] Persson R Vasilakis-Scaramozza C Hagberg KW Sponholtz T Williams T Myles P Jick SS. CPRD Aurum database: assessment of data quality and completeness of three important comorbidities. Pharmacoepidemiol Drug Saf 2020;29:1456–64. 32986901 10.1002/pds.5135

[32] Relieving pain in America: a blueprint for transforming prevention, care, education, and research. Mil Med 2016;181:397–99. 27136641 10.7205/MILMED-D-16-00012

[33] Schmidt JCF Lambert PC Gillies CL Sweeting MJ. Patterns of rates of mortality in the clinical practice research Datalink. PLoS One 2022;17:e0265709. 35925908 10.1371/journal.pone.0265709 PMC9352072

[34] Scholz SM Thalmann NF Müller D Trippolini MA Wertli MM. Factors influencing pain medication and opioid use in patients with musculoskeletal injuries: a retrospective insurance claims database study. Sci Rep 2024;14:1978. 38263185 10.1038/s41598-024-52477-7 PMC10805862

[35] Shah A Hayes CJ Martin BC. Characteristics of initial prescription episodes and likelihood of long-term opioid use—United States, 2006-2015. MMWR Morb Mortal Wkly Rep 2017;66:265–9. 28301454 10.15585/mmwr.mm6610a1 PMC5657867

[36] Shah A Hayes CJ Martin BC. Factors influencing long-term opioid use among opioid naive patients: an examination of initial prescription characteristics and pain etiologies. J Pain 2017;18:1374–83. 28711636 10.1016/j.jpain.2017.06.010 PMC5660650

[37] Stokes A Lundberg DJ Sheridan B Hempstead K Morone NE Lasser KE Trinquart L Neogi T. Association of obesity with prescription opioids for painful conditions in patients seeking primary care in the US. JAMA Netw Open 2020;3:e202012. 32239222 10.1001/jamanetworkopen.2020.2012 PMC7118518

[38] Suda KJ Durkin MJ Calip GS Gellad WF Kim H Lockhart PB Rowan SA Thornhill MH. Comparison of opioid prescribing by dentists in the United States and England. JAMA Netw Open 2019;2:e194303. 31125102 10.1001/jamanetworkopen.2019.4303 PMC6632141

[39] Swedish Council on Health Technology Assessment. SBU Systematic Review Summaries. Methods of treating chronic pain: a systematic review [internet]. Stockholm: Swedish Council on Health Technology Assessment (SBU), 2006. Copyright © 2006 by the Swedish Council on Health Technology Assessment.

[40] Taylor S Annand F Burkinshaw P Greaves F Kelleher M Knight JPC Tran A White M Marsden J. Dependence and withdrawal associated with some prescribed medicines: an evidence review. London: Public Health England, 2019. Available at: https://assets.publishing.service.gov.uk/media/5fc658398fa8f5474c800149/PHE_PMR_report_Dec2020.pdf. 10.1016/S2215-0366(19)30331-1 PMC7029276 31588045

[41] Urban H Little CB. The role of fat and inflammation in the pathogenesis and management of osteoarthritis. Rheumatology (Oxford) 2018;57(suppl_4):iv10–21. 29444323 10.1093/rheumatology/kex399

