# Supplementary material for: Clinical indications associated with new opioid use for pain management in the United Kingdom: using national primary care data
Source: Pain. 2024 Oct 24;166(3):656–66. doi: 10.1097/j.pain.0000000000003402 (PMC11808705; doi:10.1097/j.pain.0000000000003402)
Supplement: Supplementary file 1 [file jop-166-656-s001.pdf]

Supplementary Information

Supplementary Methods

Data preparation:

Drug exposure estimation using Drug Preparation Algorithm

The prescription data obtained from the Clinical Practice Research Datalink (CPRD) presents systematic challenges, such as missing information on prescription end dates and medication quantities. CPRD provides the start date of exposure in the *'issuedate'* field. However, information regarding the stop date of exposure is not directly available. Since assumptions made during this stage can have considerable implications when calculating MME/day, we employed the Drug Preparation Algorithm implemented in the R package *'drugprepr'* to estimate the stop date for opioid prescriptions.

Link to the full paper where this process was described:

https://pubmed.ncbi.nlm.nih.gov/36718594/

Numerical Daily Dose Computation

To calculate the numerical daily dose of each opioid prescription, we extracted information on **Dose Frequency** (the number of times the prescription is to be taken per day) and **Dose Number** (the number of units of drug to take at a time) from CPRD records. Plausible values were defined based on prescribing guidelines and clinical experience, with careful identification and handling of values falling outside the plausible range.

Decisions and Processing Steps in Drug Preparation Algorithm

The following decisions were made during the execution of the drug preparation algorithm to address various challenges in the data:

1. **Handle Implausible Quantities:** Set to missing.

2. **Handle Missing Quantities:** Set to the mean value for that patient for the specific product code. If missing for the entire history of the patient, set to the mean value for the entire cohort for that product code.
3. **Handle Implausible Numerical Daily Doses:** Set to missing.
4. **Handle Missing Numerical Daily Doses:** Set to the mean value for that patient for the specific product code. If missing for the entire history of the patient, set to the mean value for the entire cohort for that product code.
5. **Clean Duration:** Set to missing if the duration exceeds 6 months.
6. **Select Stop Date:** Computed as the quotient of quantity and numerical daily dose (qty/ndd).
7. **Handle Stop Date:** Set to the mean value for that product code for the individual patient.
8. **Handle Multiple Prescriptions:** Retain the record with the longest duration.
9. **Handle Overlapping Prescriptions:** Options include ignoring overlapping records.
10. **Handle Short Gaps Between Prescriptions:** Move the stop date of the preceding prescription to "fill in" the gap if the gap between consecutive prescriptions is less than 15 days, reclassifying the time as exposed.

Morphine Milligram Equivalents (MME) per day Calculation

1. **Standardization of Strength Values:** We standardized the strength values of opioid prescriptions by removing units and other measurement terms. This allowed for uniformity across different formulations.

2. **Classification of Routes of Administration:** The routes of administration were categorized into groups such as "oral," "injection," "transdermal," "buccal," etc., by standardizing the recorded routes in the dataset.
3. **Creation of Conversion Terms:** We created unique conversion terms by combining the drug substance name with its route of administration. This enabled us to apply the correct MME conversion factor for each specific opioid and formulation.
4. **Merging with Conversion Ratios:** Using a pre-existing list of MME conversion ratios we linked each opioid prescription to its corresponding MME conversion factor.
5. **Correction of Strength Values:** The strength values were corrected based on their units (e.g., mg or microgram) and adjusted for specific formulations, especially injectable and transdermal forms, to ensure accurate daily dosing calculations.
6. **Calculation of Daily Dose:** The daily dose of each opioid prescription was calculated by multiplying the corrected strength by the number of doses per day.
7. **Calculation of Daily MME:** The daily MME was obtained by multiplying the daily dose by the corresponding MME conversion factor. Special adjustments were applied for certain formulations and routes of administration to reflect accurate daily MME.
8. **Adjustments for Special Cases:** Specific adjustments were made for certain opioid formulations and routes of administration (e.g., nasal, sublingual).

Supplementary Figures

Supplementary Figure 1: Summary of methodology for generating medical codelists in CPRD AURUM for clinical indications of opioid use.

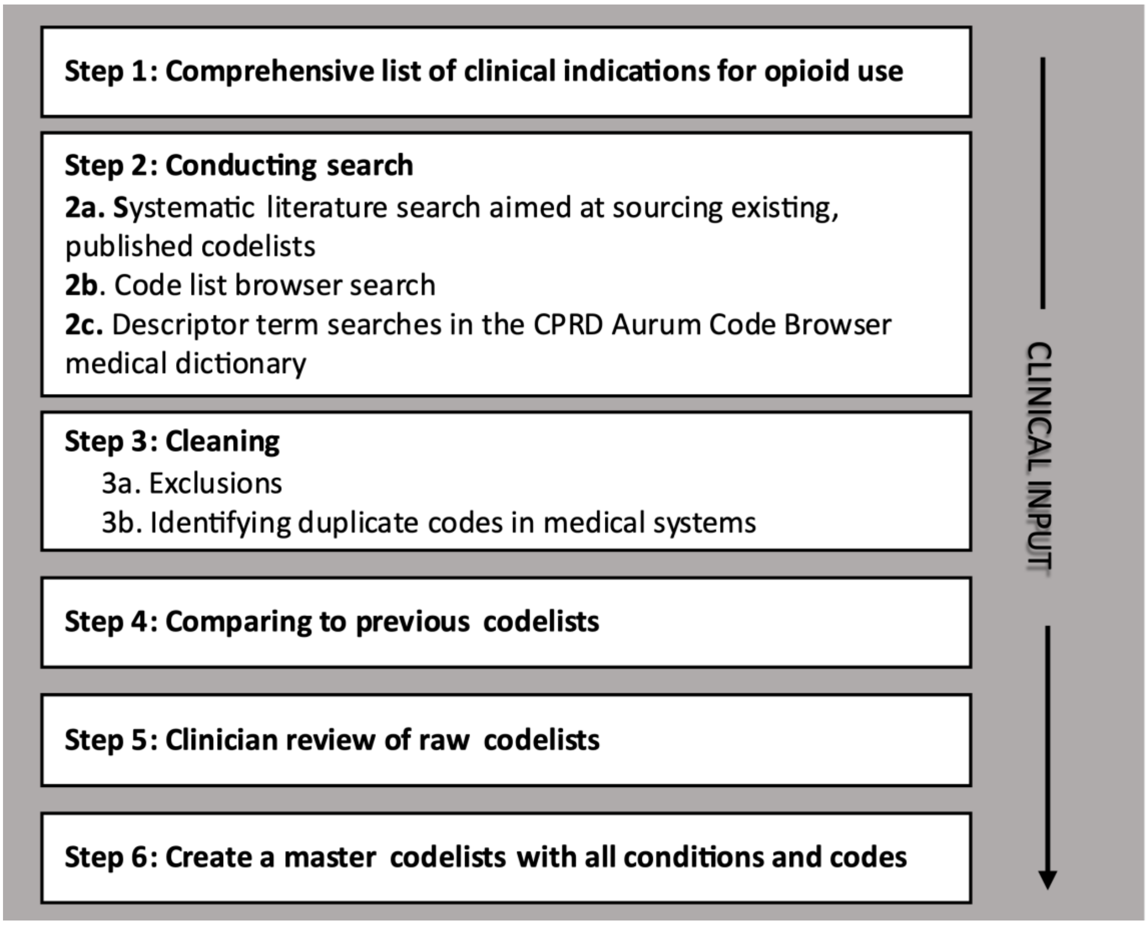

* Clinical observations are recorded in CPRD Aurum using a mixture of Read-2, SNOMED and local EMIS codes. Relevant medical codes and SNOMED-CT code lists for each clinical indication were derived using a combination of descriptor term searches against the medical dictionary available through the CPRD Code Browser Tool.

* To ensure data integrity and precision, CPRD Aurum unique identifiers, SNOMED codes, and long numeric identifiers were imported, stored, and processed as text rather than integers. The master code list was applied to the identified cohort of new opioid users without cancer to identify if they had records of surgical indications within one-year period before the index date and within five years before for chronic conditions.

Supplementary Tables

Supplementary Table 1: Patient characteristics of opioid-naïve users by clinical indication system (Full table with percentages instead of the bar chart presented in the main text).

Characteristic	Overall		Musculoskeletal		Respiratory		Infections		Neurology		Trauma / Injury		Gastrointestinal		Major Surgery		Gynaecological		Dental		Haematological	
	3,030,077		2,265,947		1,609,525		778,367		500,806		487,892		126,839		115,576		39,409		38,664		670	
**Age**																						
Mean (SD)	58	(17.1)	58.8	(16.8)	58.2	(17.2)	60	(18.2)	52.8	(17.3)	58	(18.3)	52.8	(16.3)	60.7	(16.3)	38.2	(10.5)	50.1	(17.2)	40	(13.5)
Median (IQR)	58	25	59	(24)	59	(25)	61	(27)	52	-26	58	(28)	53	(24)	64	(24)	39	(16)	49	(26)	39	(20)
**Age group (years), *n (%)***																						
18-24	80,016	3%	49,399	2%	43,113	3%	21,671	3%	21,929	4%	17,183	4%	4,386	3%	1,701	1%	5,241	13%	1,896	5%	100	15%
25-34	230,419	8%	151,983	7%	117,841	7%	57,722	7%	60,186	12%	41,052	8%	14,743	12%	8,060	7%	8,766	22%	6,633	17%	164	24%
35-44	396,133	13%	273,001	12%	206,866	13%	91,677	12%	88,692	18%	64,784	13%	22,236	18%	12,587	11%	13,304	34%	7,049	18%	163	24%
45-54	573,549	19%	425,161	19%	291,981	18%	126,936	16%	107,687	22%	87,505	18%	27,371	22%	15,019	13%	10,701	27%	7,902	20%	155	23%
55-64	629,181	21%	487,068	21%	330,624	21%	141,074	18%	89,151	18%	93,970	19%	25,244	20%	23,005	20%	1,157	3%	6,721	17%	56	8%
65-74	568,051	19%	440,942	19%	311,873	19%	147,287	19%	69,766	14%	81,810	17%	19,744	16%	30,192	26%	151	0%	4,714	12%	20	3%
≥75	552,728	18%	438,393	19%	307,227	19%	192,000	25%	63,395	13%	101,588	21%	13,115	10%	25,012	22%	89	0%	3,749	10%	12	2%
**Sex, *n (%)***																						
Male	1,191,359	39%	899,892	40%	572,186	36%	195,901	25%	138,140	28%	185,062	38%	32,351	26%	50,092	43%	20	0%	13,933	36%	217	32%
Female	1,838,689	61%	1,366,040	60%	1,037,323	64%	582,459	75%	362,662	72%	302,825	62%	94,486	74%	65,484	57%	39,389	100%	24,729	64%	453	68%
Indeterminate	29	0%	15	0%	16	0%	7	0%	4	0%	5	0%	2	0%	0	0%	0	0%	2	0%	0	0%
**MME**																						
Mean (SD)	22.3	12	23	(11.8)	22	(11.8)	22.0	(12.29)	22.4	(11.79)	23.1	(12.12)	22.8	(11.9)	28.8	(12.15)	23.3	(11.4)	23.3	(11.7)	28.4	(19.0)
Median (IQR)	19	24.8	19	(24.8)	18.9	(24.8)	18.9	(24.8)	18.9	(24.8)	18.9	(23.6)	18.9	(24.4)	29.2	(18.7)	20	(19.4)	20	(19.4)	29.2	(18)
**MME Dosage groups, *n (%)***																						
Low MME	3,016,966	100%	2,256,749	99.6%	1,603,817	100%	775,234	100%	498,896	100%	485,501	100%	126,296	100%	114,562	99%	39,251	100%	38,504	100%	644	96%
Medium MME	10,940	0%	7,800	0.3%	4,889	0%	2,485	0%	1,655	0%	2,015	0%	453	0%	901	1%	147	0%	134	0%	21	3%
High MME	1,114	0%	719	0.0%	282	0%	215	0%	120	0%	177	0%	56	0%	76	0%	6	0%	17	0%	3	0%
Very High MME	1,057	0%	679	0.0%	537	0%	433	0%	135	0%	199	0%	34	0%	37	0%	5	0%	9	0%	2	0%
**Index of Multiple Deprivation, *n(%)***																						
Least Deprived (1)	538,725	19%	411,950	19%	285,934	19%	143,891	20%	84,297	18%	91,173	20%	23,805	20%	25,077	23%	5,771	16%	6,065	17%	31	5%
2	568,096	20%	432,015	20%	301,703	20%	148,989	20%	89,813	19%	93,924	20%	24,404	20%	24,445	22%	6,474	18%	6,391	18%	35	6%
3	548,899	19%	410,493	19%	289,017	19%	139,888	19%	88,985	19%	88,297	19%	23,061	19%	21,731	20%	6,727	18%	6,970	19%	120	19%
4	589,727	21%	433,233	20%	309,111	20%	145,611	20%	98,124	21%	90,704	20%	23,143	19%	19,922	18%	8,445	23%	7,710	21%	221	36%
Most deprived (5)	617,513	22%	445,005	21%	329,194	22%	151,651	21%	107,787	23%	95,133	21%	24,907	21%	18,163	17%	9,351	25%	8,873	25%	215	35%
**Ethnicity, *n (%)***																						
White	2,246,808	87%	1,680,214	87%	1,207,966	87%	606,089	89%	362,446	83%	383,722	90%	102,458	90%	94,478	90%	27,258	79%	27,488	83%	15	3%
Asian	168,709	7%	123,164	6%	101,122	7%	42,836	6%	36,574	8%	20,590	5%	5,832	5%	5,266	5%	3,210	9%	3,068	9%	16	3%
Black	103,755	4%	76,057	4%	51,973	4%	21,465	3%	23,610	5%	12,296	3%	2,838	3%	3,112	3%	2,833	8%	1,596	5%	533	90%
Other	41,987	2%	31,162	2%	21,404	2%	9,275	1%	8,577	2%	5,000	1%	1,373	1%	1,218	1%	766	2%	562	2%	12	2%
Mixed	18,569	1%	13,653	1%	9,921	1%	4,377	1%	4,083	1%	2,718	1%	798	1%	625	1%	469	1%	306	1%	15	3%
**Drug Substance, *n(%)***																						
Codeine	2,345,268	77%	1,758,325	78%	1,252,771	78%	599,597	77%	387,130	77%	372,502	76%	96,737	76%	80,209	69%	29,767	76%	30,021	78%	390	58%
Dihydrocodeine	405,301	13%	293,669	13%	205,049	13%	95,410	12%	64,268	13%	60,963	12%	15,704	12%	15,402	13%	5,686	14%	5,006	13%	154	23%
Tramadol	202,076	7%	155,109	7%	108,190	7%	54,349	7%	36,727	7%	36,414	7%	10,948	9%	13,903	12%	3,322	8%	2,747	7%	57	9%
Morphine	35,862	1%	26,719	1%	21,290	1%	13,928	2%	5,906	1%	8,783	2%	1,595	1%	3,245	3%	326	1%	456	1%	43	6%
Buprenorphine	17,438	1%	14,623	1%	9,625	1%	6,759	1%	2,994	1%	3,935	1%	786	1%	538	0%	93	0%	193	0%	1	0%
Oxycodone	6,778	0%	5,204	0%	3,697	0%	2,147	0%	1,044	0%	1,848	0%	295	0%	1,611	1%	55	0%	70	0%	18	3%
Dextropopoxyphene/paracetamol	5,039	0%	3,282	0%	2,170	0%	1,075	0%	702	0%	713	0%	219	0%	162	0%	62	0%	40	0%	3	0%
Fentanyl	4,249	0%	3,225	0%	1,918	0%	1,517	0%	666	0%	939	0%	196	0%	203	0%	22	0%	41	0%	4	1%
Others	8,066	0%	5,791	0%	4,815	0%	3,585	0%	1,369	0%	1,795	0%	359	0%	303	0%	76	0%	90	0%	0	0%
**Missing, *n (%)***																						
Missing Ethnicity	450,249	15%	341,697	15%	217,139	13%	94,325	12%	65,516	13%	63,566	13%	10,877	9%	13,540	12%	4,873	12%	5,644	15%	78	12%
Missing IMD	167,225	6%	133,251	6%	94,566	6%	48,337	6%	31,800	6%	28,661	6%	7,519	6%	6,238	5%	2,641	7%	2,655	7%	48	7%

* Proportions are presented as percentage of non-missing data.
* Some patients had missing data for certain variables, which is reported at the end of the table.
* Other drug substances include: diamorphine, meptazinol, pethidine, tapentadol, dipipanone, alfentanil, hydromorphone, pentazocine, papaveretum and dextromoramide tartrate.
* Opioid Dosage: Low MME defined as <50 MME/day. Medium MME (50-119 MME/day), High MME (120-199 MME/day), Very High MME (>=200 MME/day).
* IQR, Interquartile range; SD, standard deviation; MME, morphine-milligram equivalent

Supplementary Table 2: Counts by Unique Patients

System	n (%)	Indications	n- New opioid users Unique patients	Percentage
		Total unique patients	**2,027,402**	100%
**Musculoskeletal**	1,639,792 (80.8%)	Osteoarthritis	1,231,972	60.7%
		Low back pain	831,983	41.0%
		MSK (non-inflammatory) bursitis, rotator cuff, tendonitis	292,755	14.4%
		Osteoporosis	96,903	4.8%
		Gout	87,440	4.3%
		Rheumatoid Arthritis	49,986	2.5%
		Fibromyalgia	48,829	2.4%
		Psoriatic arthritis	11,295	0.6%
		Systemic lupus erythematosus	6,765	0.3%
		Ankylosing spondylitis	6,690	0.3%
		Myositis	1,948	0.1%
Respiratory	1,169,042 (57.6%)	Respiratory infections	920,902	45.4%
		Cough	703,004	34.7%
		Respiratory (non-infective)	98,962	4.9%
		Fibrosis	5,965	0.3%
Infections	616,710 (30.4%)	Infections (most commonly prescribed antibiotics – UTI/cellulitis/otitis media)	615,468	30.3%
		HIV	1,756	0.1%
Trauma/ injury	413,199 (20.4%)	Trauma (including sprains, sprains, and dislocations)	228,389	11.3%
		Fractures	220,425	10.9%
Neurology	402,036 (19.8%)	Headaches (including migraine)	366,369	18.1%
		Neuropathic pain	33,843	1.6%
		Somatoform	181	<0.01%
		Demyelinating conditions (including GBS, Multiple Sclerosis)	15,432	0.7%
Major Surgery	111,345 (5.5%)	Total Knee Replacement	30,930	1.5%
		Total Hip Replacement	17,313	0.8%
		Hernia Repair	12,230	0.6%
		Hysterectomy	10,919	0.5%
		Caesarean Section	9,380	0.4%
		Cholecystectomy	7,955	0.3%
		CABG	5,783	0.%
		Vasectomy	4,033	0.20%
		Valve Replacement	3,570	0.1%
		Appendectomy	3,520	0.1%
		Prostatectomy	2,661	0.1%
		Laparoscopy	2,152	0.1%
		Limb Amputations	1,466	<0.1%
		Rotator Cuff Surgeries	1,304	<0.1%
		Carotid Endarterectomy	666	<0.1%
		Aortic Aneurysm Repair	653	<0.1%
		Resection of bladder tumour	472	<0.1%
		Mastectomy	310	<0.1%
		Lobectomy	244	<0.1%
		Resection of Prostate (including TURP)	206	<0.1%
		Lumpectomy	179	<0.1%
		Breast Reconstruction	125	<0.1%
		Aortic Root Replacement	92	<0.1%
		Pneumonectomy	12	<0.1%
Gastrointestinal	105,019 (5.18%)	IBS	88,037	4.34%
		IBD	16,993	0.84%

		Chronic Pancreatitis	1,426	0.07%
Dental	33,946 (1.67%)	Dental Pain	34,777	1.7%
Gynaecological reasons	34,777 (1.71%)	Including: (1) Dysmenorrhoea/ Endometriosis, (2) ovarian cysts, fibroids/leiomyoma (3) PID, cervicitis (4) adhesions and hydrosalpinx)	33,946	1.7%
Haematological	519 (0.03%)	Sickle Cell	519	<0.1%

Supplementary Table 4: Clinical indications of opioids with one-year look-back period (overlapping totals) – ordered by percentages for top five systems.

System	n (%)	Indications	n (%) (1 year look back)
Musculoskeletal	48.14%	Osteoarthritis	27.21%
		Low back pain	20.67%
		MSK (non-inflammatory) bursitis, rotator cuff, tendonitis	3.78%
		Rheumatoid Arthritis	1.56%
		Fibromyalgia	0.95%
		Psoriatic arthritis	0.25%
		Systemic lupus erythematosus	0.14%
		Ankylosing spondylitis	0.15%
Respiratory	24.25%	Respiratory infections	15.08%
		Respiratory (non-infective)	2.72%
		Cough	11.15%
Infections	9.87%	HIV	0.03%
		Infections (most commonly prescribed antibiotics – UTI/cellulitis/otitis media)	9.84%
Trauma/ injury	6.04%	Trauma (including sprains, sprains and dislocations)	2.96%
		Fractures	3.21%
Neurology	6.16%	Headaches	5.79%
		Neuropathic pain	0.40%
		Somatoform	0.00%

Supplementary Table 3: Neurology Clinical Indications: Demyelinating conditions (One year prior)

Condition	Frequency	n (%) (1 year look back)
Parkinson	31,467	1.04%
Multiple Sclerosis	15,582	0.51%
Motor Neurone Disease	3,581	0.12%
Guillain-Barré Syndrome	579	<0.1%
Acute Disseminated Encephalomyelitis	280	<0.1%
Transverse Myelitis	526	<0.1%
Neuromyelitis optica	102	<0.1%

Supplementary Table 4. Proportions per year for each Clinical Indication (On the Day of New Opioid Initiation)

Year	Musculoskeletal	Respiratory	Infections	Trauma	Neurology	Gastrointestinal	Major Surgery	Haematological	Gynaecological	Dental
2006	28%	4%	1%	2%	3%	0%	0%	0%	0%	0%
2007	28%	4%	1%	2%	3%	0%	0%	0%	0%	0%
2008	29%	5%	1%	2%	3%	0%	0%	0%	0%	0%
2009	30%	4%	1%	2%	3%	0%	0%	0%	0%	0%
2010	30%	4%	1%	2%	3%	0%	1%	0%	0%	0%
2011	31%	4%	1%	2%	2%	0%	1%	0%	0%	0%
2012	31%	4%	1%	2%	2%	0%	1%	0%	0%	0%
2013	31%	4%	1%	2%	2%	0%	0%	0%	0%	0%
2014	31%	4%	1%	2%	2%	0%	1%	0%	0%	0%
2015	31%	3%	1%	2%	2%	0%	1%	0%	0%	0%
2016	31%	3%	1%	2%	2%	0%	1%	0%	0%	0%
2017	32%	3%	1%	2%	2%	0%	1%	0%	0%	0%
2018	32%	2%	1%	2%	2%	0%	1%	0%	0%	0%
2019	32%	2%	1%	2%	1%	0%	1%	0%	0%	0%
2020	29%	1%	1%	2%	1%	0%	1%	0%	0%	0%
2021	28%	1%	1%	2%	1%	0%	1%	0%	0%	0%

Supplementary Table 5. Proportions per year for each Clinical Indication in a 5-year look-back period (main analysis).

Year	Musculoskeletal	Respiratory	Infections	Trauma	Neurology	Gastrointestinal	Major Surgery	Haematological	Gynaecological	Dental
2006	62%	45%	20%	16%	15%	4%	4%	0%	1%	1%
2007	65%	48%	21%	16%	15%	4%	4%	0%	1%	1%
2008	68%	50%	23%	16%	16%	4%	4%	0%	1%	1%
2009	71%	52%	24%	16%	16%	4%	4%	0%	1%	1%
2010	73%	52%	25%	16%	17%	4%	4%	0%	1%	1%
2011	74%	53%	25%	16%	17%	4%	4%	0%	1%	1%
2012	75%	54%	26%	16%	17%	4%	4%	0%	1%	1%
2013	75%	54%	26%	16%	17%	4%	4%	0%	1%	1%
2014	77%	55%	27%	16%	17%	4%	4%	0%	1%	1%
2015	78%	56%	27%	16%	17%	4%	4%	0%	1%	1%
2016	79%	56%	27%	16%	17%	4%	4%	0%	1%	1%
2017	80%	57%	28%	16%	17%	4%	4%	0%	1%	1%
2018	81%	56%	28%	16%	17%	4%	4%	0%	1%	1%
2019	83%	56%	28%	17%	17%	4%	5%	0%	1%	1%
2020	83%	55%	30%	17%	17%	4%	4%	0%	1%	1%
2021	83%	52%	30%	17%	17%	4%	4%	0%	1%	1%
